# Training through gametherapy promotes coactivation of the pelvic floor and abdominal muscles in young women, nulliparous and continents

**DOI:** 10.1590/S1677-5538.IBJU.2014.0580

**Published:** 2016

**Authors:** Valeria Regina Silva, Cássio Riccetto, Natalia Miguel Martinho, Joseane Marques, Leonardo Cesar Carvalho, Simone Botelho

**Affiliations:** 1 Departamento de Cirurgia da Faculdade de Ciências Médicas - Universidade Estadual de Campinas (UNICAMP), SP, Brasil; 2 Curso de Fisioterapia, Escola de Enfermagem, Universidade Federal de Alfenas (UNIFAL-MG), MG, Brasil

**Keywords:** Pelvic Floor, Abdominal Muscles, Electromyography

## Abstract

**Introduction and objectives:**

Several studies have been investigated co-activation can enhance the effectveness of PFM training protocols allowing preventive and therapeutic goals in pelvic floor dysfunctions. The objective of the present study was to investigate if an abdominal-pelvic protocol of training (APT) using gametherapy would allow co-activation of PFM and transversus abdominis/oblique internal (TrA/OI) muscles.

**Patients and methods:**

Twenty-five nulliparous, continent, young females, with median age 24.76 (±3.76) years were evaluated using digital palpation (DP) of PFM and surface electromyography of PFM and TrA/OI simultaneously, during maximal voluntary contraction (MVC), alternating PFM and TrA/OI contraction requests. All women participated on a supervised program of APT using gametherapy, that included exercises of pelvic mobilization associated to contraction of TrA/OI muscles oriented by virtual games, for 30 minutes, three times a week, in a total of 10 sessions. Electromyographic data were processed and analyzed by ANOVA - analysis of variance.

**Results:**

When MVC of TrA/OI was solicited, it was observed simultaneous increase of electromyographic activity of PFM (p=0.001) following ATP. However, EMG activity did not change significantly during MVC of PFM.

**Conclusion:**

Training using gametherapy allowed better co-activation of pelvic floor muscles in response to contraction of TrA, in young nulliparous and continent women.

## INTRODUCTION

Pelvic floor muscles (PFM) are responsible for urinary and fecal continence mechanisms, and participate at sexual relations and delivery (1, 2). They also are important for pelvic stabilization, along with abdominal and lumbar muscles. Some studies (3–5) have demonstrated an intimate relation between PFM and the abdomen, particularly transversus abdominis muscle (Tra) whose impact on continence mechanisms and on pelvic floor functionality is being investigated in different phases of female vital cycle. Pereira et al. (5) have identified co-activation of those muscles in young asymptomatic nulliparous women, but not among pregnant and puerperal primiparous.

It is already known that any anatomic, bio-mechanical or neuromuscular alteration can trigger functional imbalancies with consequent urogynecologic disorders (6). Dysfunctions of pelvic floor are usually multifactorial. Age, pregnancy, delivery, hormonal alterations of female cycle as well as biomechanical and postural modifications (7) can influence PFM function.

Accordingly, it is assumed that reeducation of abdominal-pelvic compartment can be beneficial to prevent and or treat female pelvic floor dysfunction (7), justifying the proposal of protocols that include abdominal muscle training, mainly Tra muscle (3, 5, 8, 9).

The objective of the present study was to identify simultaneous electrical activity of PFM and transversus abdominis/oblique internal (TrA/OI) in order to verify if a protocol of abdominal training (PAT) using gameteraphy would provide co-activation of those muscles.

## METODOLOGY

### Study type

Prospective, clinical study.

### Sample

From January to June 2014, 25 young nulliparous continent women were recruited (median age 24.7±3.7 years) through an informative lecture at Physiotherapy and Nurse schools of the Federal University of Alfenas - UNIFAL/MG. The study was approved by the ethical committee of the University of Campinas Medical School - UNICAMP (CAAE protocol: 19625113.5.0000.5404), and all participants signed a free consent form according to Helsinki Declaration. The study was authorized to be realized at the UNIFAL-MG.

### Inclusion criteria

Nulliparous young female were included, with 18 to 35 years old, without any micturition complaints (score zero according to Portuguese validated question form International Consultation on Incontinence Questionnaire Short Form -ICIQ UI-SF) (10).

### Exclusion criteria

Exclusion criteria included virgin women (impossibility to apply electromyographic evaluation with endovaginal sensors); previous abdominal-pelvic surgeries; metabolic disorders (high blood pressure and diabetes); presence of myopathies and collagen diseases, neurologic alterations, cognitive disturbance and physical limitations that prevented participation; previous PFM training (supervised by a health professional); grade zero contractility of PFM, according to the Modified Scale of Oxford (11), without evident contraction of PFM.

### Evaluation procedures

The study was performed by two investigators (VS and JM). Evaluations and revaluations were performed by a single researcher (JM) and the training protocol was applied by the main author (VS) who was unaware of the clinical conditions of the participants.

The participant was held in orthostatic position and the abdominal region was cleaned with 70% alcohol; adherent and dischargeable sensors were positioned above the topography of TrA/OI muscles (2cm away from the antero-superior iliac spine towards pubis). The participants were instructed to correctly contract TrA/OI during expiratory phase, in dorsal decubitus with inflected inferior limbs.

Evaluation of PFM was initially performed by digital palpation, in order to graduate contractility according to Modified Scale of Oxford (11) and to orientate the participant on how to effectively contract PFM. Participants were asked to contract PFM while the evaluator pushed the fingers cranially during expiratory phase (12, 13) avoiding the use of gluteus and adductor muscles (5).

Electromyographic activity of PFM and TrA/OI was recorded using an EMG equipment (EMG System do Brasil^®^), consisting of a signal conditioner with a filter with frequencies of 20-500HZ, amplifier of 1000x and rejection of common proportion of >120Db. Also, a conversion plate of A/D signal of 12 bit was used to convert analogic signs to digital signs, with sample frequency of filter 2.0khz and entrance band of 5mv. All data were transmitted in microvolts (μv) to the equipment software (AqData^®^) connected to a notebook processed in Root Mean Square (RMS) making sure that all electric equipments were turned off from electric network during collection of data (5).

PFM EMG was recorded using an endovaginal sensor (Physio-Med Services^®^), manually introduced by the researcher with the aid of a hypoallergenic gel, positioned at the lateral wall of the vagina. Reference electrode was positioned at the right fist (between the radius and the styloid process of the ulna) (14).

Electromyographic evaluation protocol consisted on the recording of simultaneous collection of PFM (channel 1) and TrA/OI (channel 2), at rest, for 15 seconds, in order to use them during normalization of the electromyographic data, followed by three MVC (maximal voluntary contraction) of PFM, with simultaneous record of electromyographic response of TrA/OI.

After that, three MVC of TrA/OI with simultaneous recording of PFM response were performed. Each MVC was performed following a rest period of three minutes in order to avoid muscular fatigue (14).

### Abdominal - pelvic training program

The protocol consisted of ten individual sessions of 30 minutes, supervised by the main investigator (physiotherapist) three times a week.

**Figure 1:**
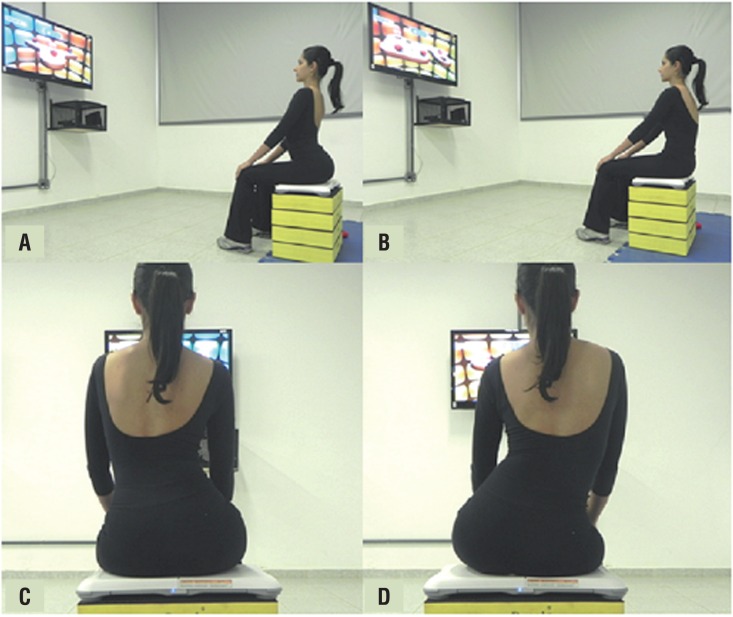
Movements are performed over the platform, during virtual games. Anteversion movement (A), retroversion (B), lateral pelvic inclination (D)

The exercises were performed emphasizing the abdominal-pelvic compartment using virtual games. This protocol was based on the work proposed by Martinho (2014), (15–17). It was used the Wii™ console and the game Wii Fit Plus™, using the sub-games: Lotus Focus, Penguin Slide, Table Tilt and Balance Bubble. The protocol was developed in order that the participant played seated on a Wii Balance Board platform positioned over an adjustable bench, allowing the knees and hips to form a flexion angle of 90o. In order to execute the games, many pelvic exercises that demanded trunk control using the abdominal muscles (TrA/OI) without active contraction of PFM were used (anteversion, retroversion, lateral pelvic inclination) (Figure-1).

### Data processing and analysis

**Figure 2:**
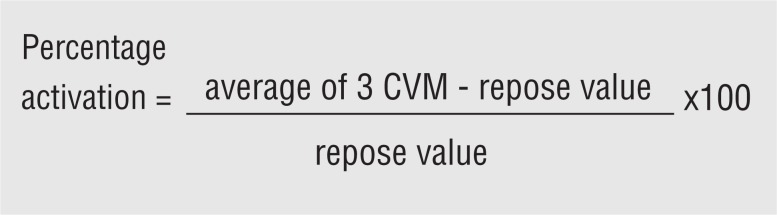
Calculus of percentage of variation in relation to rest.

Initially, five seconds of each MVC were selected, considering the medium of three RMS (expressed in μv) for each participant. In order to investigate simultaneous electromyographic activity of PFM and TrA/OI (co-activation) it was calculated the percentage variation of activation related to rest of booth muscles, according to the following formulae (Figure-2):

Statistical analysis was performed using ANOVA (analysis of variance), using the software SAS System for Windows (Statistical Analysis System), version 9.2. SAS Institute Inc, 2002-2008, Cary, NC, USA. Significance level was set at 5%.

## RESULTS

Table-1 presents clinical and social-demographic characteristics of participants.

**Table 1:** Social-demographic and clinical characteristics of participants.

Social-demographic characteristics		
Color of the skin* (%)		
	White	92
	Non white	8
Schooling* (%)		
	Complete/incomplete higher education	100
Marital status* (%)		
	Single	84
	Married/stable union	16
Work** (%)		
	No labor	76
	Labor	24
Income revenue* (%)		
	1-2 times a week	16
	3-4 times a week	20
	>4 times a week	64
**Clinical characteristics**		
**Age (years) (M±sD)**		24.76
		(3.76)
**Body Mass Index (Kg/m^2^) (M±sD)**		22.34
		(3.70)
**Physical activity (%)**		
	Sedentaries	52
	Active	48
**Sexual activity (%)**		
	Absent	32
	Present	68
**Stool movements (%)**		
	Less than 3 times a week	20
	Higher than 3 times a week	80

During evaluation of PFM contractility using digital palpation, it was observed that most women presented contractions grade 3 or 4 according to Modified Scale of Oxford (11) (Table-2).

**Table 2:** Evaluation of contractility of PFM by digital palpation, before and after training.

Modified Scale of Oxford	Before training (f-%)	After training (f-%)	Time P-value*
1	1 (4)	0 (0)	
2	4 (16)	2 (8)	
3	13 (52)	13 (52)	0.0001
4	7 (28)	9 (36)	
5	0 (0)	1 (4)	

Table presents distribution of participants according to Modified Scale of Oxford (presented data in absolute frequency-fe percentage-%) and comparison between the time of evaluation before and after training.

* Wilcoxon test.

The main objective of this study was to investigate the presence of co-activation of PFM and TrA/OI before and after a program of ATP, using gametherapy. Table-3 present the results showing an increase of co-activation of PFM when it was solicited a maximal voluntary contraction of TrA/OI after training.

**Table 3:** Co-activation of muscles in response to maximal voluntary contraction, before and after training.

	Before training	After training	Time P-value*
Co-activation PFM (MVC TrA/OI)	127.27	147.84	0.01
Co-activation TrA/OI (MVC PFM)	234.19	196.72	0.1

Table presents muscular response (co-activation) following MVC of PFM or TrA/OI, comparing the time of evaluation before and after training. Values expressed in percentage (5).

Note increase of co-activation of PFM when MVC was solicited to TrA/OI

**PFM** = pelvic floor muscles

**MVC** = maximal voluntary contraction

**TrA/OI** = transversus abdominis/oblique internal

* **ANOVA** for repeated measures with transformation by posts.

Power of sample: 0.06

## DISCUSSION

PFM training has been recommended to prevent and treat female pelvic floor dysfunctions since 1948, when Arnold Kegel (18) introduced the practice of repeatedly and singly contract those muscles.

Historically, PFM training programs oriented women to not contract abdominal, gluteus and adductor muscles, for those were considered accessory muscles (19). Until now, few anatomic and functional studies (20, 21) showed the true relation among muscles that form abdominal-pelvic compartment.

According to Piret and Beziers (2002) (20), transversus abdominis muscle is inserted in the same layer of transverse muscle of perineum. Delancey et al. (2004) (21) reported that in normal women increase of abdominal pressure promotes contraction of elevator anus muscle diminishing the genital hiatus. On the other hand, Junginger et al. (2010) (22) observed that bladder neck is elevated only when PFM contractions are higher than intra-abdominal pressure.

Caufriez (1997) (23) developed the hypo-pressure gymnastics technique that stimulates the recruitment of PFM following activation of abdominal muscles associated to diaphragmatic aspiration. But only after the studies of Sapsford & Hodges (2001) (8) the investigation of the relationship of those muscles were intensified and demonstrated that there is a co-activation of PFM during electrical activity of Tra (3–5). Neumman and Gil (2002) (3) showed that relaxing abdominal muscles prevents efficient contraction of PFM, suggesting a strong relationship among them.

Stupp et al. (2011) (24) in order to investigate if hypo-pressure gymnastic technique could trigger activation of both muscles-PFM and TrA-showed that MVC of TrA and PFM simultaneously is as efficient as isolated contraction of PFM.

In the present study, when co-activation of those muscles was analyzed, it was observed a significant increase of electrical activity of PFM following training, when MVC of TrA/OI was solicited. One of the hypothesis to explain that fact is the solicitation of maintenance of TrA contraction during the execution of exercises induced by virtual games, favoring co-activation of PFM; the performance of exercises in a virtual environment allows the participant to interact and feedback the real time activities. In this context, the use of game-therapy is been quite explored as biofeedback for physiotherapy treatment (15–17).

Kamel et al. (2012) (25) proposed training of abdominal muscles and observed significant improvement of PFM pressure evaluated by vaginal perineometer, suggesting indirect action of abdominal muscles on PFM activation, providing coordination, support and resistance. Rogers (2008) (26) described improvement of PFM perception following a training program justifying increase of co-activation of PFM.

On the other hand, in the present study it was not observed significant co-activation of TrA during MVC of PFM. Similar results were presented by Perschers et al. (2001) (27). Some factors could influence muscle synergy such as position of evaluation, which normally is different than the adopted position of daily activities, as well as the influence of posture in the order of muscular activation, also cited by Madill (2009) (28).

Specially in nulliparous young women, Pereira et al. (2013) (5) observed significant co-activation of both TrA/OI and PFM when MVC was solicited to both; however, co-activation was not observed in pregnant and puerperal women, suggesting the existence of other factors that influence the behavior of those muscles.

The study has some limitations, such as the small sample and reduced number of sessions. In spite of the fact that there is no consensus in literature regarding the ideal time of training to improve PFM functionality, Bø et al. (1990) (29) suggest improvement following six months of training.

Most studies investigate the effects of training in general in symptomatic women. Very few information is known regarding the pattern of muscular behavior of asymptomatic young women, who do not suffer interference of age, hormonal alterations, obesity, pregnancies and deliveries, as well of urogynecological signals and symptoms, one of the most important aspects of the present study.

Also, it is difficult to stablish in which condition it is more probable to observe positive results: while treating young asymptomatic women, with more probability of “normal” muscular performance or those with urogynecological symptoms who respond to treatment. These aspects can influence treatment adherence and follow-up. In our study we observed good adherence. Participants reported satisfaction with the training, for it was innovative and stimulated PFM. No side effect was reported after training.

One of the challenges in this area is to introduce these trainings involving abdominal muscles for prevention, particularly PFM and TrA, in order to prevent overload of pelvic floor during daily activities. Pre-contraction of these muscles during daily activities that involve increase of intra-abdominal pressure (for example, sports or gymnastic) may be fundamental to prevent future dysfunctions, with improvement of quality of life and consequent reduction of treatment costs (30). Other studies must be performed in young and healthy populations to elucidate the effects of different kinds of training on anatomic and functional aspects of that population.

## CONCLUSIONS

Abdominal-pelvic training using gametherapy improved co-activation of pelvic floor muscles in response to contraction of transversus abdominis and oblique internal, in young, continent nulliparous women.

## ABBREVIATIONS

ANOVA: = Analysis of variance
MVC: = Maximal Voluntary Contraction
SP: = Standard Deviation
EMG: = Surface Electromyography
ICIQ-UI SF: = International Consultation on Incontinence Questionnaire Short-Form
PVM: = Pelvic Floor Muscles
OI: = Oblique Internal
RMS: = Root-mean-square
APT: = Abdomino-pelvic Training
TrA: = Transversus abdominis
μV: = microvolts
